# Perfluorooctane Sulfonate Disturbs Nanog Expression through miR-490-3p in Mouse Embryonic Stem Cells

**DOI:** 10.1371/journal.pone.0074968

**Published:** 2013-10-01

**Authors:** Bo Xu, Xiaojiao Chen, Zhilei Mao, Minjian Chen, Xiumei Han, Guizhen Du, Xiaoli Ji, Chunxin Chang, Virender K. Rehan, Xinru Wang, Yankai Xia

**Affiliations:** 1 State Key Laboratory of Reproductive Medicine, Institute of Toxicology, Nanjing Medical University, Nanjing, China; 2 Key Laboratory of Modern Toxicology of Ministry of Education, School of Public Health, Nanjing Medical University, Nanjing, China; 3 State Key Laboratory of Reproductive Medicine, Nanjing Maternity and Child Health Hospital, Nanjing Medical University, Nanjing, China; 4 Department of Pediatrics, Los Angeles Biomedical Research Institute at Harbor-UCLA Medical Center at David Geffen School of Medicine, Torrance, California, United States of America; University of Colorado, Denver, United States of America

## Abstract

Perfluorooctane sulfonate (PFOS) poses potential risks to reproduction and development. Mouse embryonic stem cells (mESCs) are ideal models for developmental toxicity testing of environmental contaminants *in vitro*. However, the mechanism by which PFOS affects early embryonic development is still unclear. In this study, mESCs were exposed to PFOS for 24 h, and then general cytotoxicity and pluripotency were evaluated. MTT assay showed that neither PFOS (0.2 µM, 2 µM, 20 µM, and 200 µM) nor control medium (0.1% DMSO) treatments affected cell viability. Furthermore, there were no significant differences in cell cycle and apoptosis between the PFOS treatment and control groups. However, we found that the mRNA and protein levels of pluripotency markers (Sox2, Nanog) in mESCs were significantly decreased following exposure to PFOS for 24 h, while there were no significant changes in the mRNA and protein levels of Oct4. Accordingly, the expression levels of *miR-145* and *miR-490-3p,* which can regulate Sox2 and Nanog expressions were significantly increased. Chrm2, the host gene of *miR-490-3p*, was positively associated with *miR-490-3p* expression after PFOS exposure. Dual luciferase reporter assay suggests that *miR-490-3p* directly targets Nano*g*. These results suggest that PFOS can disturb the expression of pluripotency factors in mESCs, while *miR-145* and *miR-490-3p* play key roles in modulating this effect.

## Introduction

Perfluorooctane sulfonate (PFOS) has been widely used as a surface-active agent for a wide range of commercial, industrial and household applications, including water repellents, lubricants, paints, and fire-fighting foams [1]. It has been identified in various environmental sectors, including air [2], sewage sludge [3], [4], snow, lake, and surface runoff water [5]. PFOS is also commonly detected in maternal serum, amniotic fluid [6], umbilical cord blood [7], breast milk [8], nail, hair and urine [9] and semen [10]. PFOS is a kind of persistent lipophilic compound which exhibited high degrees of bioconcentration from water and biomagnification from food [11], [12]. As it has been shown to bind strongly to plasma albumin [13], there is a high accumulation of PFOS in humans, so it has a long half-life in serum (5.4 y) [14]. In light of its environmental persistence, bioaccumulation, and potential toxicity, PFOS exposure generates great concern about its potential impacts on health. There is a large body of evidence to support potential adverse effects of PFOS on development in humans and animals. Epidemiology studies have found that exposure to PFOS is correlated with reduced birth weight [15], [16], motor or mental developmental milestones in early childhood [17]. Even in non-human primates, PFOS exposure has been shown to cause decreased body weights [18]. In addition, PFOS exposure can induce neonatal death [19], [20], [21], delayed growth and development, and delayed eye opening in rodents [20], [22], [23]. In aquatic models, such as zebrafish and medaka, PFOS-induced abnormalities have been observed. Exposure to PFOS could alter immunoregulation functions in fish larvae, impact F1 offspring morphology, behavior, and survival in zebrafish [24], [25], and result in a decrease in hatch time and hatch rate [26], [27]. Although numerous studies have suggested the developmental toxicity of PFOS, little is known about the underlying molecular mechanisms.

Mouse embryonic stem cells (mESCs), derived from inner cell mass of preimplantation blastocysts, while propagating in pluripotency state, maintain the capacity to generate any cell type in the body. As the existing toxicity assays using fully differentiated cell lines or immortal cell lines can’t reflect a series of stages during the embryonic development, mESCs may be an ideal model for in vitro testing safety or toxicity of chemicals and environmental contaminants. Elucidation of the transcriptional regulatory circuitry operating in ES cells is fundamental for understanding the molecular mechanisms of pluripotency.

Many studies have demonstrated that microRNAs (miRNAs) played important roles in development. Mice without miRNAs die at embryonic day 7.5 [28]. miRNAs are required for the formation of many tissues, such as the vertebrate limb [29], skin [30], and the lung epithelium [31]. miRNAs are also important components of the transcriptional regulatory networks and these have emerged as central players in the maintenance of ESC self-renewal and differentiation [32], [33], [34]. They may offer a mean to direct the differentiation of ES cells into desired fates and inhibit the formation of undesired lineages, such as the cardiac differentiation [35], and neural differentiation [36].

In this study, to better understand the effects and the molecular mechanisms of PFOS on early embryonic development, we tested the effects of PFOS on general cytotoxicity and pluripotency of mESCs, and further explored the role of miRNAs in PFOS-induced effects.

## Materials and Methods

### Chemicals and Reagents

PFOS (≥98% purity), dimethyl sulfoxide (DMSO), bovine serum albumin (BSA), diethylpyrocarbonate (DEPC), 3-(4,5-dimethylthiazol-2-yl)-2,5-diphenyl tetrazolium bromide (MTT) were obtained from Sigma-Aldrich (St. Louis, MO, USA). Stock solution of PFOS was dissolved in DMSO at a concentration of 200 mM, stored at −20°C, and then diluted to desired concentrations in culture medium immediately before use. The final concentration of DMSO in the culture medium did not exceed 0.1%. All chemicals were of analytical grade.

### Cell Culture and PFOS Treatment

Mouse ES cell line D3 [American Type Culture Collection (ATCC), Manassas, VA, USA, no.CRL-11632] was kindly provided by Stem Cell Bank, Chinese Academy of Sciences. This cell line has been widely used in previous studies [37], [38]. mESCs were grown on mouse embryonic fibroblast feeder cells (MEF) that were treated by mitomycin C in knock-out Dulbecco’s modified Eagle’s medium (Gibco BRL, Grand Island, NY) supplemented with 20% ES qualified fetal bovine serum (Gibco BRL), 0.1 mM β-mercaptoethanol (Sigma Chemical, St Louis, MO), 0.1 mM nonessential amino acids (Gibco BRL), 0.1 mM L-glutamine (Gibco BRL), 0.1 mM pyruvate sodium, 100 unit/ml penicillin/streptomycin (Gibco BRL) and 1000 U/ml of leukemia inhibitory factor (LIF) (Millipore, Billerica, MA). Fresh medium was changed every day, and cells were passaged every 3 days at 37°C and 5% CO_2_. Cells were dislodged using trypsin/EDTA (Gibco BRL). Before the start of experiments, feeder cells were depleted by incubating trypsinized cells in complete ES cells medium on cell culture dishes for 30 min, during which time feeder cells attached to the dish while mESCs not. The mESCs were treated with PFOS (0.2 µM, 2 µM, 20 µM, and 200 µM) dissolved in DMSO. Cells were exposed to 0.1% DMSO as a negative control.

Human 293T cells were obtained from ATCC (Manassas VA, USA) and cultured in complete growth medium DMEM (Hyclone, UT, USA), supplemented with 10% fetal bovine serum (10% FBS), 100 U/mL penicillin, and 100 µg/mL streptomycin at 37°C, 5% CO_2_.

### Cell Viability Assay, Morphological Study and Alkaline Phosphatase Staining

The feeder depleted ES cells were seeded on gelatin coated plates at a density of about 1.5×10^4^ per well in 96-well plates and 1×10^6^ per well in 6-well plates and incubated overnight. ES cellular viability was evaluated using the MTT proliferation assay. MTT (5 mg/ml) was dissolved in PBS, sterilized by filtration through a 0.22 µm Millipore® filter and stored at 4°C. After exposure to PFOS at different concentrations, the cells were washed twice with PBS. Then 25 µl of MTT were added to each well, and the cells were incubated for 4 h at 37°C to allow MTT metabolism. The medium was replaced with 150 µl DMSO, plates were shaken for 10 min, and the absorbance was determined at 490 nm. Results were presented as percentage of the control.

The morphology of cells was examined and recorded using phase contrast microscope (Olympus, CK41, Japan) after exposure to PFOS (0.2 µM, 2 µM, 20 µM, and 200 µM) or control medium (0.1% DMSO). Alkaline phosphatase (AP) staining was performed with the Alkaline Phosphatase Complete Kit (Sidansai Corporation of Biotechnology, Shanghai, China) according to the manufacturer’s protocol. The cells were washed twice with PBS and fixed for 2 min with fixing solution at room temperature. The cells were washed with PBS and incubated with AP staining solution for 30 min, protected from light. After being washed with PBS, the cells were photographed using a microscope (Olympus, CK41, Japan).

### Cell Cycle Analysis and Apoptosis Assay

To determine if PFOS could affect the cell cycle and induce apoptosis of mESCs, flow cytometric analysis was used to determine the state of cell cycle and apoptosis. Feeder depleted ES cells were seeded on gelatin-coated 6-well plates at a density of about 1×10^6^ cells per well. Cells were incubated overnight and subsequently exposed to PFOS (0.2 µM, 2 µM, 20 µM, and 200 µM) and 0.1% DMSO. After 24 h, cells were washed with PBS and harvested with trypsin/EDTA. Cells were fixed in 75% ethanol for 2 h or washed in cold PBS, then stained with propidium iodide (PI) and annexin V for 30 min protected from light. The fixed/stained cells were analyzed by FACS Calibur Flow Cytometry (BD Biosciences, NJ, USA) to quantify cell cycle or cell apoptosis.

### RNA Isolation and Quantitative Real-Time PCR Assay

Total RNA was isolated using TRIZOL reagent (Invitrogen, Carlsbad, CA) according to the manufacturer’s instructions, and the concentration of total RNA was determined by measuring the absorbance at 260 nm. cDNA synthesis for coding genes and miRNAs were performed with 1 µg of total RNA according the manufacturer’s instructions (Takara, Tokyo, Japan).

### mRNA (Oct4, Sox2, Nanog, Chrm2, GAPDH) and miRNAs

The expression of miRNAs (*has*/*mmu-miR-145*, *has*/*mmu-miR-490-3p*, U6) were analyzed using SYBR PCR Master Mix reagent kits (Takara) according to the manufacturer’s instructions. Primer sequences are shown in Table S1. All oligonucleotide primers were synthesized by Invitrogen (Shanghai). All real-time PCR reactions were carried out on ABI7900 Fast Real-Time System (Applied Bio systems, Foster City, CA, USA) according to the manufacturer’s instructions. All experiments were repeated at least three times.

### Western Blot Analysis

The total cellular proteins were extracted using radio immunoprecipitation assay (RIPA) buffer containing protease inhibitors (Complete, Roche, Basel, Switzerland). Protein concentrations were determined using bicinchoninic acid (BCA) Protein Assay kit (Biyuntian, China). Equal amounts of protein (60 µg) from each sample that solubilized in the sample buffer (25 mM Tris, pH 6.8, 1% SDS (w/v), 5% β-mercaptoethanol(v/v), 1 mM EDTA, 4% glycerol, and 0.01% bromophenol blue) were fractionated by electrophoresis on a 12.3% polyacrylamide-SDS gel at 90 V for 3 h. The proteins were then transferred to a polyvinyldiene difluoride membrane (PVDF, Bio-Rad, Hercules, CA). The membrane with transferred proteins was incubated in buffer containing specific rabbit polyclonal antibodies for Sox2/Nanog or goat polyclonal antibodies for Oct4 (Abcam, Kendall square, MA, USA, 1∶1000 dilution), followed by incubating with goat anti-rabbit or donkey anti-goat secondary antibody conjugated with horseradish peroxidase at 1∶1000. The specific signals were detected by the enhanced chemiluminescence (ECL Western blotting detection reagents, Amersham Life Science Limited). The amount of GAPDH (34 kDa) in each lane was used as a loading control for the amount of Oct4 (45 kDa), Sox2 (43 kDa), or Nanog (35 kDa). All experiments were repeated at least three times. Blots were quantified by densitometry and normalized by the use of GAPDH to correct for differences in loading of the proteins. For densitometric analyses, the bands on the blots were measured by Eagle Eye II Still Video Imaging System (Stratagene, La Jolla, CA).

### Bioinformatics: Predict Potential miRNAs, mRNA

miR-145 has been previously identified as targeting Sox2 in hESC [39]. We filtered the microRNA.org and identified *miR-490-3p* as targeting Nanog. Cholinergic muscarinic receptor 2 (Chrm2), as the host gene of *miR-490-3p*, was identified by miRBase [40].

### Transfection and Dual-luciferase Reporter Gene Assay

Synthetic miRNA precursor molecules of *miR-490-3p*, a negative control, *miR-490-3p* inhibitor and a inhibitor control (GenePharma, Shanghai, China) were used in transfection experiments. Human 293T cells were cultured to about 50% confluence and transfection was carried out using Lipofectamine 2000 (Invitrogen Corp, CA, USA) with 50 nM *miR-490-3p* mimics, a negative control,100 nM *miR-490-3p* inhibitor or a inhibitor control in 6-well plates respectively. After 24 h of transfection, total RNA was isolated from the transfected cells. The 3′UTR sequence of Nanog predicted to interact with *miR-490-3p* or a mutated sequence with the predicted target sites were inserted into the KpnI and SacI sites of pGL3 promoter vector (Genscript, Nanjing, China). These constructs were named pGL3-Nanog*-miR 490-3p*-WT, pGL3-Nanog*-miR 490-3p*-Mut. For reporter gene assay, cells were plated onto 24-well plates and transfected with 800 ng of the pGL3- Nanog*-miR-490-3p*-WT, pGL3-Nanog*-miR-490-3p*-Mut, 50 nM *miR-490-3p* mimics and control respectively. A Renilla luciferase vector pRL-SV40 (5 ng) was also co-transfected to normalize the differences in transfection efficiency. Cells were collected at 24 h posttransfection and luciferase assays were performed with a dual-luciferase reporter system (Promega, Madison, WI) according to the manufacturer’s instructions. Firefly luciferase activity measured was normalized to Renilla luciferase activity. Transfection was repeated three times in triplicate.

### Data Analysis

Statistical analysis was performed using STATA9.2 (Stata CorpStata Corp, LP). Values are expressed as means ±S.E. for all experiments. Statistically significant differences between the treatments and the control were examined by one-way ANOVA, followed by Dunnett’s multiple comparison test. We used the method of 2^−ΔΔCt^ to analyze the results of RT-PCR. All tests of statistical significance were two-sided, and the statistical significance was set at p<0.05.

## Results

### Effects of PFOS on Cell Viability, Morphology and Alkaline Phosphatase Staining in mESCs

**Figure 1:**
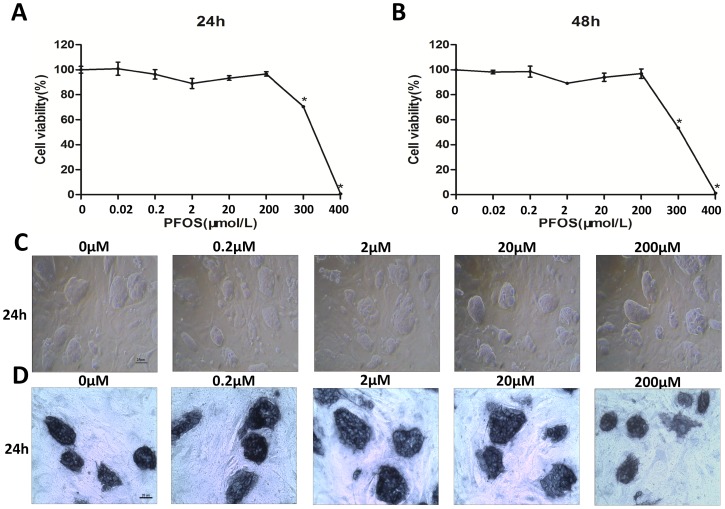
Effects of PFOS on cell viability and morphology in mESCs. (A and B) Cell viability was determined by MTT assay after exposure to various concentrations of PFOS for 24 h and 48 h. (C) D3 mESCs was exposed to PFOS for 24 h, cell morphology was observed. Magnification, 100×. Cells were cultured with various concentrations of PFOS (0.2 µM, 2 µM, 20 µM, and 200 µM) or DMSO as control. (D) Effects of PFOS on alkaline phosphatase staining in mESCs. Cells were cultured with various concentrations of PFOS (0.2 µM, 2 µM, 20 µM, and 200 µM) or DMSO as control. Scale bar = 25 µm. Values of the experiment were represented as the percentages of cell viability compared with that of the control and expressed as means ± S.E. from five separate experiments in which treatments were performed in quadruplicate. *indicates significant difference when the values were compared to that of the control at *p<0.05*.

To identify the effects of PFOS on cell viability and morphology, D3 mESCs were exposed to various concentrations of PFOS for 24 h and 48 h. As shown in Figure 1A, 1B. PFOS treatment significantly affected cell viability at 300 µM and 400 µM doses. After 24 h of treatment with PFOS, the morphology of mESCs was similar to that of control cells treated with DMSO (Figure 1C). To determine whether PFOS influences pluripotency, we stained mESCs with alkaline phosphatase (AP). We observed that mESCs treated with PFOS for 24 h did not appear differentiated and were similar to the control cells with deep staining and full colony morphology (Figure 1D). Since there is no difference in cytotoxic effects between 24 h treatment and 48 h treatment, in all of the following experiments, cells were exposed to PFOS for 24 h.

### Effects of PFOS on Cell Cycle and Apoptosis in mESCs

We examined the effects of PFOS on the cell cycle and apoptosis after 24 h exposure by flow cytometery. We found no significant difference in cell cycle between treatment groups and the control group (Figure S1A and S1B), nor were there any significant difference in apoptosis between treatment groups and the control group (Figure S1C and S1D).

### Effects of PFOS on the Potential and the Relative Expression of *miR-145 and miR-490-3p* in mESCs

**Figure 2:**
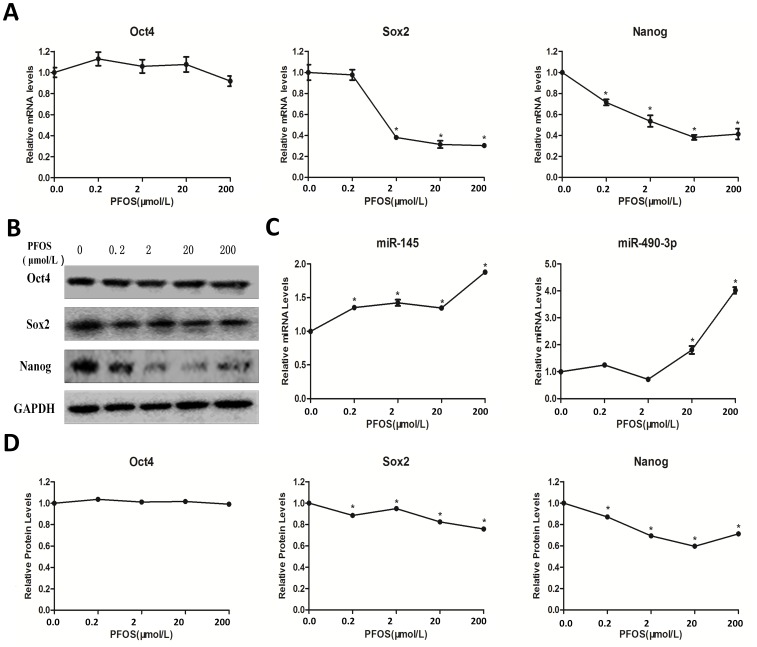
Effects of PFOS on pluripotency and expressions of *miR-145, miR-490-3p* in mESCs. Cells were cultured with various concentrations of PFOS (0.2 µM, 2 µM, 20 µM, and 200 µM) or DMSO as control for 24 h. (A) Oct-4/Sox-2/Nanog mRNA levels were determined by quantitative real-time PCR using a housekeeping gene GAPDH as an internal control. (B) The protein levels of Oct-4/Sox-2/Nanog were determined by Western blot analysis using GAPDH as an internal control. (C) miRNA levels(*miR-145*, *miR-490-3p)* were determined by quantitative real-time PCR and were normalized to U6 as an internal control. Each data point was normalized to the control (DMSO) and represented the means ± S.E. from three independent experiments. (D) Relative protein levels of Oct4, Sox2 and Nanog. *indicates significant difference when the values were compared to that of the control (*p<0.05*).

We detected the effects of PFOS on the potential by examining the expression of self-renewal factors (Oct4, Sox2, Nanog). Exposure to PFOS in mESCs significantly decreased expression of Sox2, Nanog, at both mRNA and protein levels. However, the mRNA and protein levels of Oct4 were unchanged (Figure 2A, Figure 2B).

To explore the mechanisms by which PFOS disturbed the observed altered expression of Sox2, Nanog in mESCs, the expressions of *miR-145* and *miR-490-3p* which may target Sox2, Nanog were evaluated. We found that both *miR-145* and *miR-490-3p* were dose dependently increased after PFOS exposure.

### The Expression Chrm2 after PFOS Exposure

**Figure 3:**
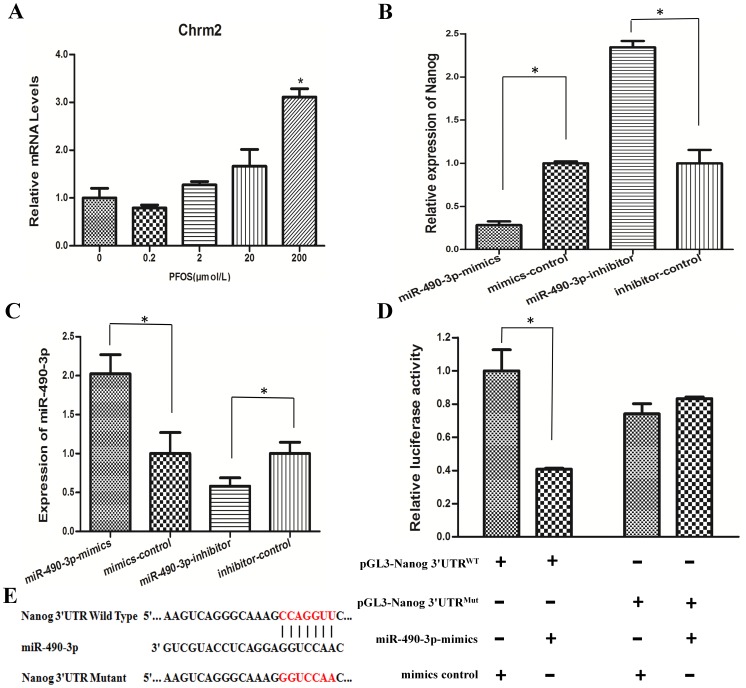
Over-expression of *miR-490-3p* reduced Nanog expression. (A)The expression of its host gene Chrm2 mRNA levels was determined by quantitative real-time PCR using a housekeeping gene GAPDH as an internal control. Cells were cultured with various concentrations of PFOS (0.2 µM, 2 µM, 20 µM, and 200 µM) or DMSO as control for 24 h. (B) Cells were transfected with 50 nM miR-490 mimics or 100 nM miR-490 inhibitor for 24 h. qRT-PCR was performed to evaluate the mRNA level of Nanog. (C) The relative expression levels of *miR-490-3p* after transfection. (D) Cells were co-transfected with *miR-490-3p* mimics and negative control, renilla luciferase vector pRL-SV40 and Nanog 3′UTR luciferase reporters for 24 h. Both firefly and Renilla luciferase activities are measured in the same sample. Firefly luciferase signals were normalized with Renilla luciferase signals. (E) Sequence alignment of miR-490-3p with 3′ UTR of Nanog. Bottom: mutations in the 3′UTR of Nanog in order to create the mutant luciferase reporter constructs. *indicates significant difference compared with that of control cells (P<0.05). All tests were performed in triplicate and presented as means±SE. Reporter activity was significantly decreased after miR-490-3p overexpression compared to control (P<0.05).

We compared the expression levels of *miR-490-3p* host gene Chrm2 after PFOS exposure by qRT-PCR (Figure 3A). The expressions of Chrm2 were increased. In order to explore a potential relationship between *miR-490-3p* and Chrm2, the Pearson correlation analysis was performed. A significantly positive correlation was found between the expression levels of *miR-490-3p* and Chrm2 (R^2^ = 0.7902, p<0.001. Figure S2), indicating that *miR-490-3p* was transcribed together with its host gene Chrm2.

### Transfection and Dual-luciferase Reporter Gene Assays

We predicted *miR-490-3p* might be the potential miRNA for targeting Nanog mRNA, and our results showed an increase of *miR-490-3p* and a corresponding decrease of Nanog expression after PFOS exposure, To further validate the hypothesis that *miR-490-3p* regulates Nanog expression after PFOS exposure, we transfected *miR-490-3p* mimics and a negative control precursor, *miR-490-3p* inhibitor and a inhibitor control precursor in 293T cells. The mRNA and protein levels of Nanog were evaluated after transfection for 24 h. As expected, qRT-PCR analysis showed that the relative expression levels of Nanog mRNA was decreased with *miR-490-3p* mimics and Nanog mRNA was increased with *miR-490-3p* inhibitor in 293T cells (Figure 3B). To confirm the efficiency of transfection assay, the miRNA expression level of *miR-490-3p* was measured after transfection with *miR-490-3p* mimics and a negative control precursor, *miR-490-3p* inhibitor and a inhibitor control precursor. The results showed that the relative expression levels of *miR-490-3p* were increased with *miR-490-3p* mimics and that of *miR-490-3p* expression levels were decreased with *miR-490-3p* inhibitor in 293T cells (Figure 3C).

To investigate whether *miR-490-3p* directly bind to the 3′UTR regions of Nanog, we performed miRNA dual luciferase reporter assay by constructing the wild type and mutant type luciferase reporter plasmids containing the binding region of the 3′UTR of Nanog mRNA. We found that co-transfection of *miR-490-3p* mimics and pGL3-Nanog 3′UTR reporter plasmids significantly decreased the luciferase activity in 293T cells, as compared with the control (Figure 3D, Figure 3E). These results suggested that *miR-490-3p* could directly target Nanog.

## Discussion

Previous reports have shown that PFOS was associated with developmental toxicity. In this study, we analyzed the effects of PFOS exposure on pluripotency of mESCs. Results showed that PFOS exposure didn’t cause any changes in morphology and AP staining of mESCs, but resulted in downregulation of pluripotency markers (Sox2, Nanog) expression both at the mRNA and protein levels, which agreed with the fact that EtOH, 17β-Estradiol, 4-tert-octylphenol (OP) and 4-nonylphenol (NP), did not change AP activity in mESCs, but affected the expression of some pluripotency factors [41], [42].

Nanog, the natural killer-2 class homeobox transcription factor, can control a cascade of pathways, including pluripotency, self-renewal, genome surveillance and cell fate determination [43]. Nanog can also sustain pluripotency in ES cells even in the absence of LIF [44]. Knockdown of Nanog can induce differentiation to extraembryonic endoderm and trophectoderm lineages in Human ESCs [45]. Nanog deficient mESCs lose pluripotency and differentiate into extraembryonic endoderm lineage [46]. Sox2, the SRY family member, can promote the expression of ESC-specific genes and suppress differentiation in the transcriptional network [47]. It is indispensable for maintaining ESC pluripotency. Sox2-null ES cells can differentiate primarily into trophoectoderm-like cells [48]. Collectively, both Sox2 and Nanog are essential in the maintenance of ESC self-renewal and pluripotency. The expression level of Nanog was decreased after exposure to 0.2 µM PFOS in mESCs, and 0.2 µM can represent occupational exposure to PFOS [49]. Moreover, it is reported that exposure to PFOS(10^−7^ M) is correlated with motor or mental developmental milestones in early childhood in humans [17]. Similarly, the expression level of Sox2 was decreased at 2 µM, and 2 µM is aquatic environmentally relevant. There is also a report indicating that PFOS(10^−6^ M) exposure can induce toxicity in zebrafish [50]. Therefore, the biological significance of our findings regarding the effect of low-dose PFOS on the expression of Sox2, Nanog should be taken into consideration.

The discovery of miRNAs provides an new layer for gene regulation, and miRNAs are thought to be functionally important in regulating the self-renewal and pluripotency of ESCs [51], [52]. Previous studies indicate that miRNAs control the expression of pluripotency factors [53], [54]. By using bioinformatic software (microRNA.org), we predicted that *miR-490-3p* might be the potential miRNA for targeting Nanog mRNA. By combing the results obtained from transfection and dual luciferase reporter assay, we firstly confirmed that miR-490-3p regulated the expression of Nanog in mESCs, which provided a new insight into PFOS-induced toxicity in mESCs. miR-145 had been previously identified as targeting Sox2 in hESCs [39]. As *miR-145* is homologous miRNA in both of human and mouse, so it may also play an important role in modulating mESCs pluripotency through its ability to target and regulate the expression of Sox2 in mESCs.

While cytoxicity was observerd in Vero cells [55], neonatal gonocyte and Sertoli cells [56], ESCs-derived cardiomyocytes [57], Neural stem cells [58], Cerebellar granule cells [59], human adrenocortical carcinoma [60] after PFOS exposure (<100 µM), the morphology and AP staining of mESCs were unchanged up to 200 μΜ of PFOS. However, as we mentioned above, the expression level of Nanog was decreased at 0.2 µM and the expression level of Sox2 was decreased at 2 µM. Therefore, at relatively low doses, although PFOS didn’t alter the phenotype of mESCs, the gene alterations have occurred, which may be due to unlimited proliferation and high self-renewal of mESCs.

## Conclusions

The current study revealed that PFOS exposure could decrease the expression of Nanog and Sox2 in mESCs. In addition, our findings showed that *miR-490-3p* could directly target Nanog. Meanwhile, results here also suggested that PFOS could affect the expression of Chrm2, which might, at least in part, modulate *miR-490-3p* expression. These findings allow us to conclude that *miR-490-3p* and its host gene Chrm2 regulate Nanog expression in mESCs, providing novel insights into the molecular mechanisms of developmental toxicity of PFOS.

## Supporting Information

Figure S1 **Effects of PFOS on cell cycle and apoptosis in mESC.** Cells were cultured with various concentrations of PFOS (0.2 µM, 2 µM, 20 µM, and 100 µM) or DMSO as control for 24 h. The cell cycle and apoptosis were analyzed by flow cytometry. 10,000 cells were analyzed for each sample. (A and B) The pictures of cell cycle were shown in (A). Data of the experiment was expressed as a percentage of total cells. Results quantitated in cell cycle were shown in (B). (C and D) Cells in the LL quadrant indicated that they were live cells. Cells in the LR quadrant were in the early stages of apoptosis. Cells in the UR quadrant were late apoptotic (C). The percentage of apoptotic cells was also presented in histogram (D). Each data point was represented as the means ± S.E. from three separate experiments in which treatments were performed in triplicate.
(TIF)

Figure S2 **Correlation between the levels of** ***miR-490-3p*** **and** ***Chrm2*** **by Pearson correlation analysis.** (R^2^ = 0.7902, p<0.001).
(TIF)

Table S1 **Sequences of primers for qRT-PCR.**
(DOCX)
