# Correction: Perfluorooctane Sulfonate Disturbs Nanog Expression through miR-490-3p in Mouse Embryonic Stem Cells

**DOI:** 10.1371/journal.pone.0221486

**Published:** 2019-08-16

**Authors:** Bo Xu, Xiaojiao Chen, Zhilei Mao, Minjian Chen, Xiumei Han, Guizhen Du, Xiaoli Ji, Chunxin Chang, Virender K. Rehan, Xinru Wang, Yankai Xia

In Fig 2b, the image appearing in the GADPH row is incorrect. Please see the complete, correct Fig 2 here.

**Fig 2:**
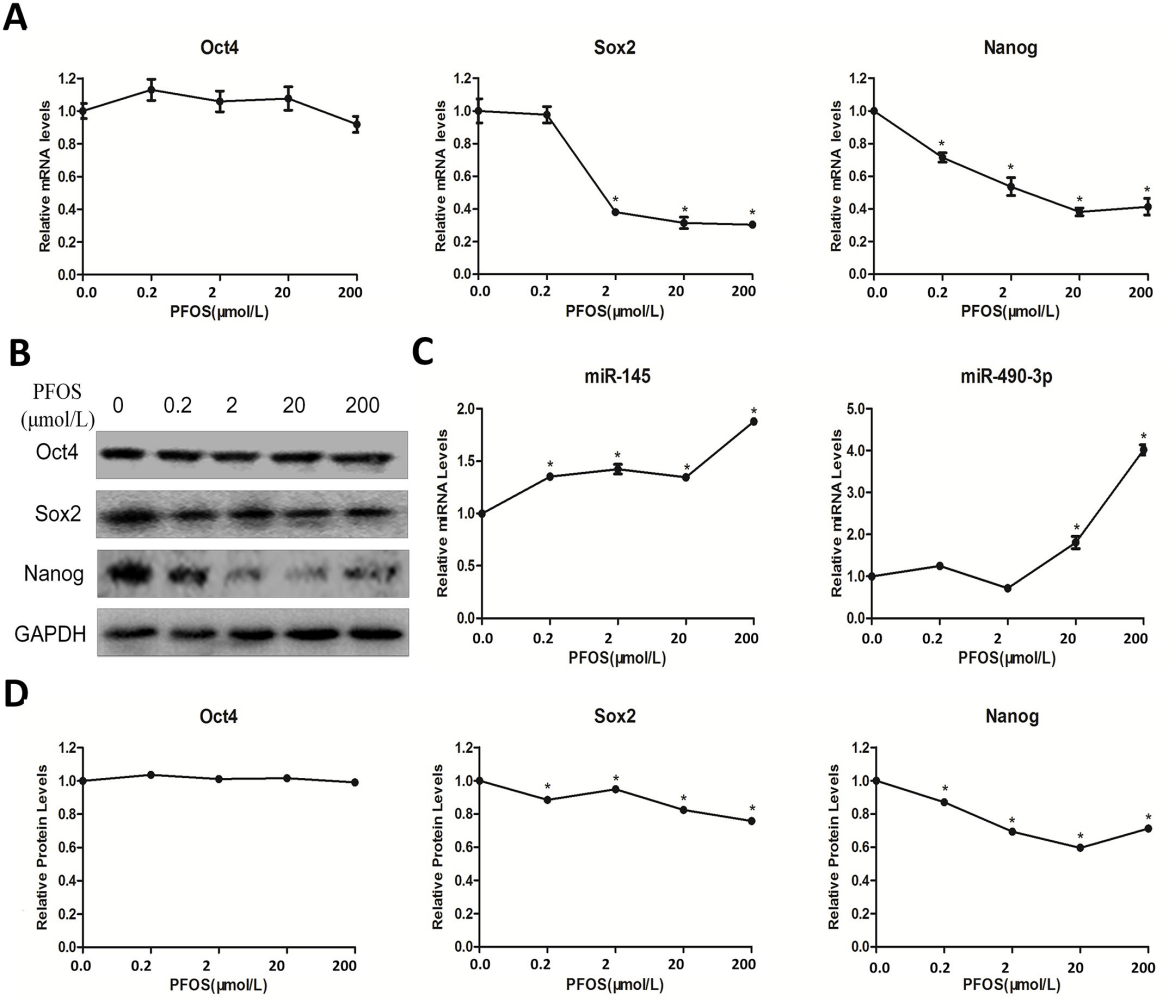
Effects of PFOS on pluripotency and expressions of *miR-145*, *miR-490-3p* in mESCs. Cells were cultured with various concentrations of PFOS (0.2 μM, 2 μM, 20 μM, and 200 μM) or DMSO as control for 24 h. (A) Oct-4/Sox-2/Nanog mRNA levels were determined by quantitative real-time PCR using a housekeeping gene GAPDH as an internal control. (B) The protein levels of Oct-4/Sox-2/Nanog were determined by Western blot analysis using GAPDH as an internal control. (C) miRNA levels (*miR-145*, *miR-490-3p*) were determined by quantitative real-time PCR and were normalized to U6 as an internal control. Each data point was normalized to the control (DMSO) and represented the means ± S.E. from three independent experiments. (D) Relative protein levels of Oct4, Sox2 and Nanog. *indicates significant difference when the values were compared to that of the control (*p<0*.*05*).
