# Supplementary figures and images for: Perfluorooctane Sulfonate Disturbs Nanog Expression through miR-490-3p in Mouse Embryonic Stem Cells
Source: PLoS One. 2013 Oct 1;8(10):e74968. doi: 10.1371/journal.pone.0074968 (PMC3788095; doi:10.1371/journal.pone.0074968)

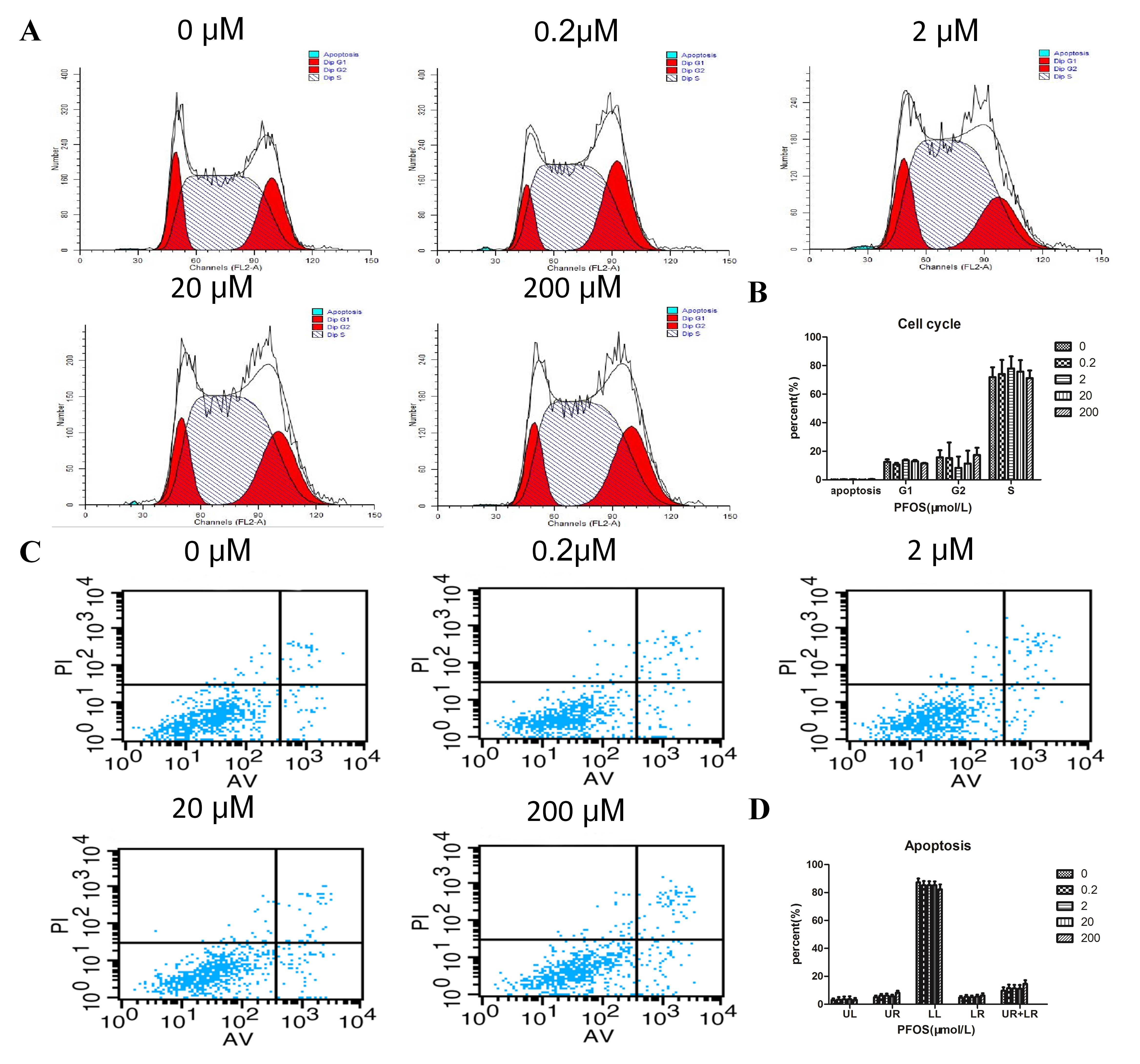

Supplement: Figure S1 — Effects of PFOS on cell cycle and apoptosis in mESC. Cells were cultured with various concentrations of PFOS (0.2 µM, 2 µM, 20 µM, and 100 µM) or DMSO as control for 24 h. The cell cycle and apoptosis were analyzed by flow cytometry. 10,000 cells were analyzed for each sample. (A and B) The pictures of cell cycle were shown in (A). Data of the experiment was expressed as a percentage of total cells. Results quantitated in cell cycle were shown in (B). (C and D) Cells in the LL quadrant indicated that they were live cells. Cells in the LR quadrant were in the early stages of apoptosis. Cells in the UR quadrant were late apoptotic (C). The percentage of apoptotic cells was also presented in histogram (D). Each data point was represented as the means ± S.E. from three separate experiments in which treatments were performed in triplicate. (TIF) [file pone.0074968.s001.tif]

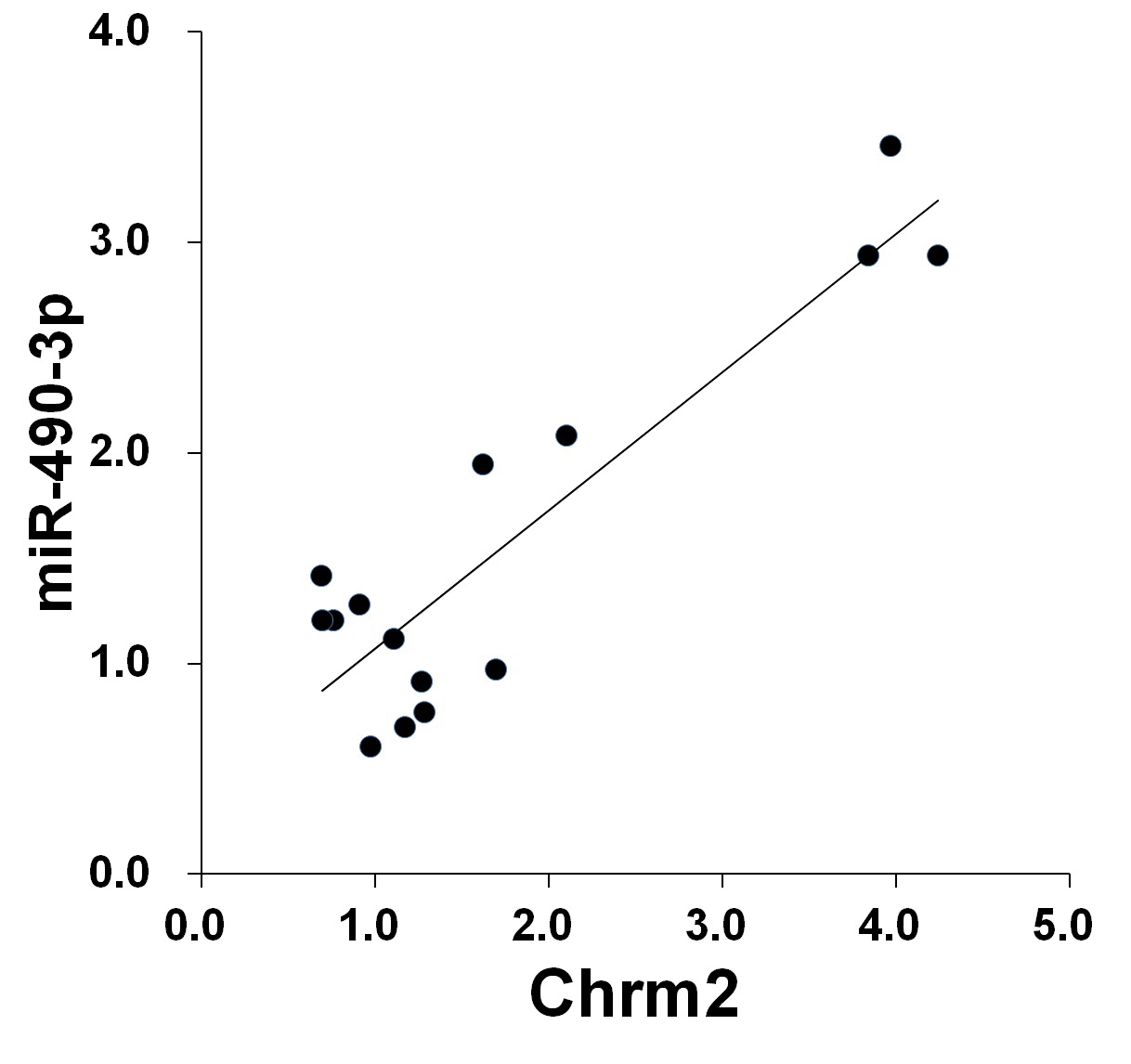

Supplement: Figure S2 — Correlation between the levels of miR-490-3p and Chrm2 by Pearson correlation analysis. (R2 = 0.7902, p<0.001). (TIF) [file pone.0074968.s002.tif]
