# Supplementary material for: Perfluorooctane Sulfonate Disturbs Nanog Expression through miR-490-3p in Mouse Embryonic Stem Cells
Source: PLoS One. 2013 Oct 1;8(10):e74968. doi: 10.1371/journal.pone.0074968 (PMC3788095; doi:10.1371/journal.pone.0074968)
Supplement: Table S1 — Sequences of primers for qRT-PCR. (DOCX) [file pone.0074968.s003.docx]

Table S1. Sequences of primers for qRT-PCR.

| *miRNA* |  | Sequences |
| --- | --- | --- |
| *hsa/mmu-miR-145* | Reverse | 5'-CTCAACTGGTGTCGTGGAGTCGGCAATTCAGTTGAGAGGGATTC-3’ |
|  | Forward Primer | 5'-ACACTCCAGCTGGGGTCCAGTTTTCCCAGGA-3’ |
|  | Reverse Primer | 5'-CTCAACTGGTGTCGTGGAGTC-3’ |
| *hsa/mmu-miR-490-3p* | Reverse | 5'-CTCAACTGGTGTCGTGGAGTCGGCAATTCAGTTGAGCAGCATGG-3’ |
|  | Forward Primer | 5'-ACACTCCAGCTGGGCAACCTGGAGGACTCC-3’ |
|  | Reverse Primer | 5'-CTCAACTGGTGTCGTGGAGTC-3’ |
| *U6* | Reverse | 5'-AACGCTTCACGAATTTGCGT-3’ |
|  | Forward Primer | 5'-CTCGCTTCGGCAGCACA-3’ |
|  | Reverse Primer | 5'-TGGTGTCGTGGAGTCG-3’ |
| *hsa/mmu-miR-490-3p mimics* | Sense | 5'-CAACCUGGAGGACUCCAUGCUG-3’ |
|  | Anti-sense | 5'-GCAUGGAGUCCUCCAGGUUGUU-3’ |
| *mimics control* | Sense | 5'-UUCUCCGAACGUGUCACGUTT-3’ |
|  | Anti-sense | 5'-ACGUGACACGUUCGGAGAATT-3’ |
| *hsa/mmu-miR-490-3p inhibitor* | Sense | 5'-CAGCAUGGAGUCCUCCAGGUUG-3’ |
| *inhibitor control* | Sense | 5'-CAGUACUUUUGUGUAGUACAA-3’ |

| Gene |  | Sequences |
| --- | --- | --- |
| *mmu-Oct4* | Forward Primer | 5'-GCCCTCCCTACAGCAGATCA-3’ |
|  | Reverse Primer | 5'-GAACCATACTCGAACCACATCCTT-3’ |
| *mmu-Sox2* | Forward Primer | 5'-AAGACGCTCATGAAGAAGGATAAGTAC-3’ |
|  | Reverse Primer | 5'-CGCTCGCCATGCTGTTC-3’ |
| *mmu-Nanog* | Forward Primer | 5'-TGCTGAGCCCTTCTGAATCA-3’ |
|  | Reverse Primer | 5'-TCCATTCTGAACCTGAGCTATAAGC-3’ |
| *hsa-Nanog* | Forward Primer | 5'-TCCCGAGAAAAGATTAGTCAGCA-3’ |
|  | Reverse Primer | 5'-AGTGGGGCACCTGTTTAACTT-3’ |
| *mmu-Chrm2* | Forward Primer | 5'-CTAACCTACCCAGTTAAGCGGA-3’ |
|  | Reverse Primer | 5'-TGACGGCAGCGTTGGAAAA-3’ |
| *mmu-GAPDH* | Forward Primer | 5'-AGGTCGGTGTGAACGGATTTG-3’ |
|  | Reverse Primer | 5'-GGGGTCGTTGATGGCAACA-3’ |
| *hsa-GAPDH* | Forward Primer | 5'-GGAGCGAGATCCCTCCAAAAT-3’ |
|  | Reverse Primer | 5'-GGCTGTTGTCATACTTCTCATGG-3’ |
